# Promoting helpful attention and interpretation patterns to reduce anxiety and depression in young people: weaving scientific data with young peoples’ lived experiences

**DOI:** 10.1186/s12888-021-03320-0

**Published:** 2021-08-25

**Authors:** Jennifer Y. F. Lau, Rebecca Watkins-Muleba, Isabelle Lee, Victoria Pile, Colette R. Hirsch

**Affiliations:** 1 Psychology Department, IOPPN, King’s College London, Denmark Hill, London, SE5 8AF UK; 2 Youth Resilience Research Unit, Queen Mary University of London, London, UK; 3 Royal Holloway University of London, London, UK

**Keywords:** Adolescence, Affective disorders, Information-processing biases, Prevention

## Abstract

**Background:**

Anxiety and depression are common, disabling and frequently start in youth, underscoring the need for effective, accessible early interventions. Empirical data and consultations with lived experience youth representatives suggest that maladaptive cognitive patterns contribute to and maintain anxiety and depression in daily life. Promoting *adaptive* cognitive patterns could therefore reflect “active ingredients” in the treatment and/or prevention of youth anxiety and depression. Here, we described and compared different therapeutic techniques that equipped young people with a more flexible capacity to use attention and/or promoted a tendency to positive/benign (over threatening/negative) interpretations of uncertain situations.

**Methods:**

We searched electronic databases (PubMed, PsycINFO, EMBASE, and PsycARTICLES) for studies containing words relating to: intervention; youth; anxiety and/or depression and attention and/or interpretation, and selected studies which sought to reduce self-reported anxiety/depression in youth by explicitly altering attention and/or interpretation patterns. Ten young people with lived experiences of anxiety and depression and from diverse backgrounds were consulted on the relevance of these strategies in managing emotions in their daily lives and also whether there were additional strategies that could be targeted to promote adaptive thinking styles.

**Results:**

Two sets of techniques, each targeting different levels of responding with different strengths and weaknesses were identified. Cognitive bias modification training (CBM) tasks were largely able to alter attention and interpretation biases but the effects of training on clinical symptoms was more mixed. In contrast, guided instructions that teach young people to regulate their attention or to evaluate alternative explanations of personally-salient events, reduced symptoms but there was little experimental data establishing the intervention mechanism. Lived experience representatives suggested that strategies such as deliberately recalling positive past experiences or positive aspects of oneself to counteract negative thinking.

**Discussion:**

CBM techniques target clear hypothesised mechanisms but require further co-design with young people to make them more engaging and augment their clinical effects. Guided instructions benefit from being embedded in clinical interventions, but lack empirical data to support their intervention mechanism, underscoring the need for more experimental work. Feedback from young people suggest that combining complimentary techniques within multi-pronged “toolboxes” to develop resilient thinking patterns in youth is empowering.

## Background

Anxiety and depression are common, disabling and frequently start in youth, underscoring the need for effective, accessible early interventions. Identifying the “active ingredients” of therapeutic change (factors, which when targeted, contribute to symptom reduction) can improve existing treatments and/or develop novel mechanism-based interventions. Equipping individuals with a more flexible capacity to use attention (enabling shifts of attention towards positive over threatening/negative information depending on circumstances) and a tendency to endorse positive/benign (over threatening/negative) interpretations of uncertain situations – could reflect active ingredients [1].

**Fig. 1:**
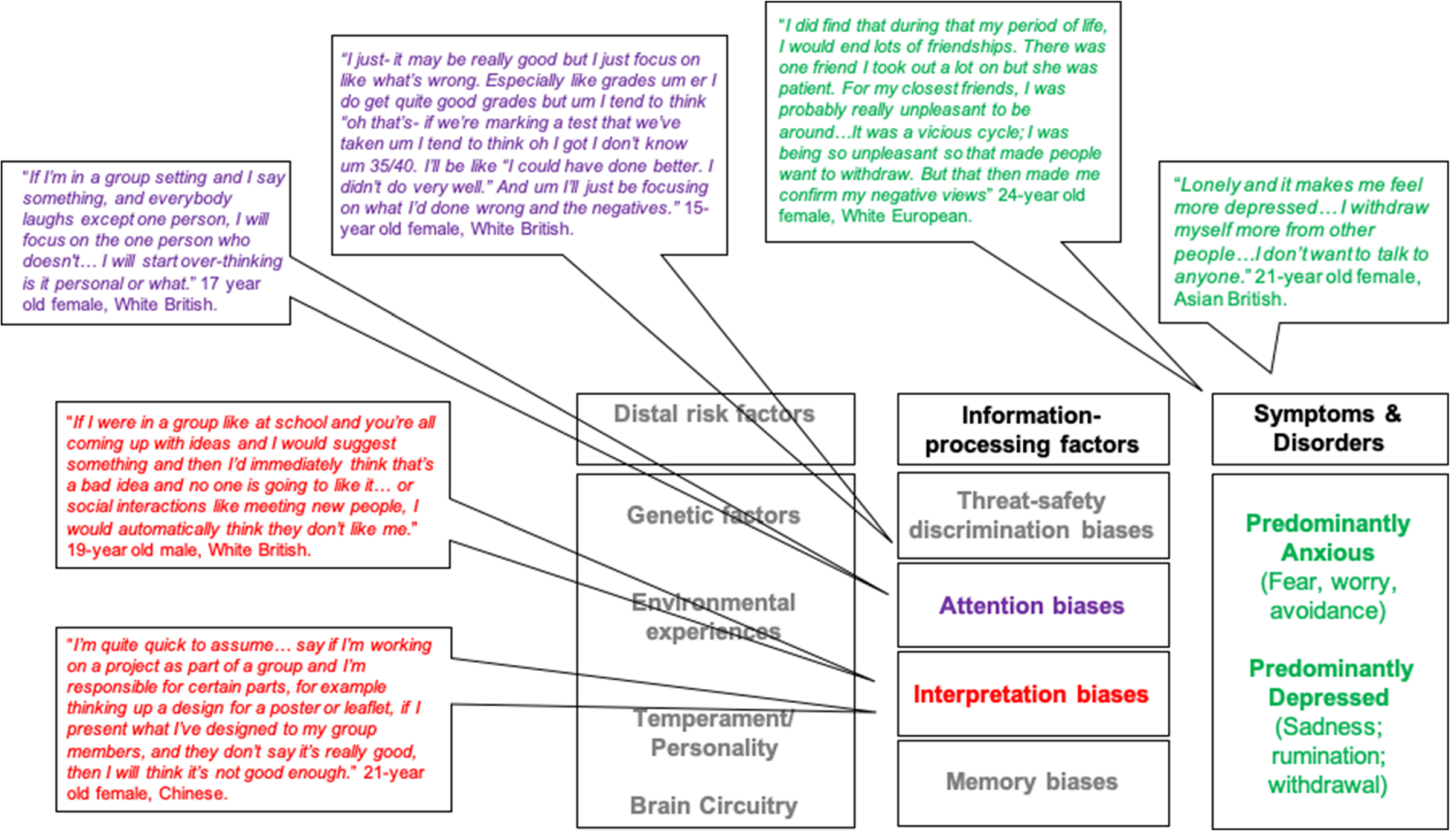
An expanded information processing account of youth anxiety and depression [1] corroborated by young peoples’ experiences. Information-processing biases and the role of distal factors that are beyond the scope of this report are greyed out

This suggestion draws on two evidence strands. Key cognitive theories propose that maladaptive attention and interpretation patterns (those focusing on or favouring threatening/negative information) maintain and increase risk for anxiety and depression [2]. Developmental cognitive neuroscience data support these theories in youth [1]. Moreover, consultations with lived experience youth representatives further corroborated the role of these cognitive factors in real-life mood fluctuations (Fig. 1), motivating and reinforcing this research focus. Second, adult treatment models of anxiety and depression show that challenging maladaptive attention and interpretation patterns and encouraging more resilient patterns is efficacious [3]. As youth involves significant growth in relevant emotion regulation abilities, adopting resilient cognitive patterns, before maladaptive ones become habitual, is fruitful [4].

Yet, outstanding questions remain on how we can most effectively target attention and interpretation patterns and whether alteration of these factors can reduce symptoms in potential treatment and prevention contexts. In this review, we describe interventions that explicitly target each cognitive patterns and compare their effectiveness at reducing symptoms in those with clinical anxiety/depression and those displaying high symptoms levels. Where applicable, we describe pre-existing individual differences that moderate symptom reduction effects. We explore whether combined interventions targeting both processes yield greater therapeutic effects [5, 6]. Finally, we consult lived experiences youth representatives over their experiences of using helpful thinking patterns in daily life, their effectiveness and how these techniques could be further enhanced.

## Method

### Systematic review

Our review was pre-registered at PROSPERO (CRD42020196651). Following published guidelines for the Preferred Reporting Items for Systematic Reviews and Meta-Analyses (PRISMA) [7], in June/July 2020, we searched electronic databases (PubMed, PsycINFO, EMBASE, and PsycARTICLES) for studies containing words relating to: intervention; youth; anxiety and/or depression and attention and/or interpretation. Titles, abstracts and where required, full texts were screened to determine inclusion in the review by two psychology students (IL, RWM), with a smaller subset (10%) reviewed by the lead author JL. We applied the following inclusion criteria: 1) peer-reviewed original research published in English-language journals; 2) investigated human participants with a mean age between 14.0 and 24.9 years; 3) reported on interventions or manipulations that sought to reduce self-reported anxiety/depression by explicitly altering attention and/or interpretation patterns.

For each paper, we recorded: publication details, participant numbers and demographics in the intervention and comparison group, primary presenting problem (anxiety/depression/both) and how this was assessed (diagnostic interview/questionnaire), and the intervention and comparison condition characteristics (dosage/delivery mode). Effect sizes of any pre-to-post anxiety/depression symptom change in the intervention group and the between-group post-intervention symptom difference between intervention and comparison group participants were included/calculated. Heterogeneity across study samples, designs, and intervention characteristics warranted a narrative synthesis rather than meta-analyses.

### Consultations with lived experience representatives

Ten young people (aged 15 to 24 years; 7 females; 6 White British) with varying severity of past anxiety and depression (and treatments) were invited to provide consultation about the relevance of managing unhelpful thinking styles in daily life. Efforts were taken to encourage diversity in age range, gender and ethnicity.

Young people were invited through online advertisements on dedicated research websites. A topic guide was developed for consultation sessions, which occurred as one-to-one meetings with the researchers (JL, VP). This topic guide asked young people to think about situations that provoked feelings of anxiety and depression, the thinking patterns that amplified negative emotions, and strategies they took to challenge unhelpful thinking patterns. Young peoples’ perspectives on how thinking patterns could amplify negative emotions are presented in Fig. 1 and served as a rationale for our systematic search. However, their thoughts on strategies used to challenge unhelpful thinking patterns were used to complement the findings of our systematic search.

## Results

**Fig. 2:**
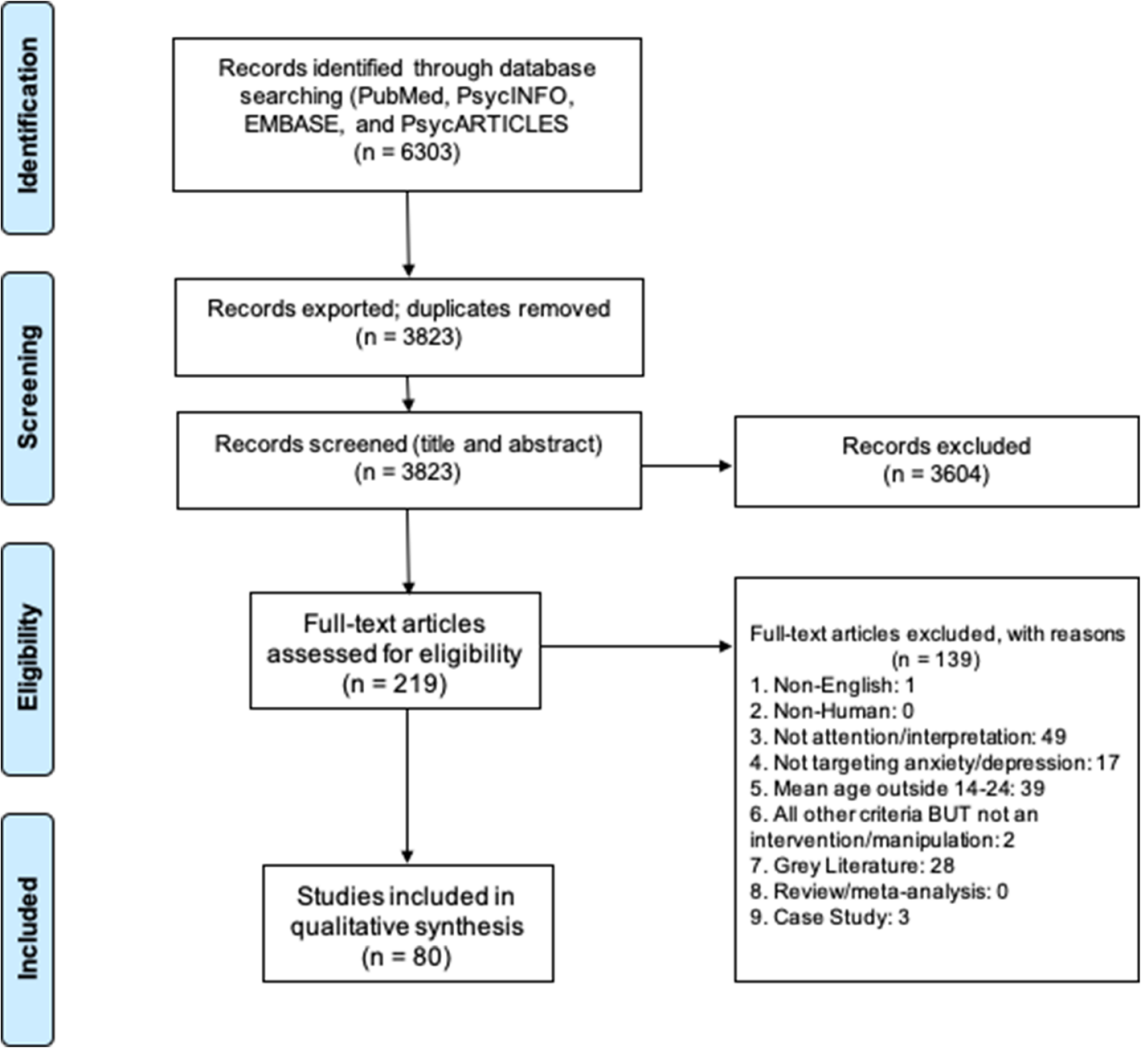
Diagram showing selection of studies

Study selection is summarised in Fig. 2. Of the final 80 papers, 22 studies modified attention patterns; 52, interpretational patterns; and 6, targeted both. All studies were used to address the range and effectiveness of interventions targetting attention and interpretational patterns. To address symptom reduction effects, only studies involving “clinical” participants (those reporting diagnoses of anxiety/depression) and “high-symptom” participants (those selected on the basis of symptoms above a clinical cut-off or in relation to other participants) were used. For these studies, we only included those that involved at least two intervention sessions as many early studies (even involving clinical/high-symptom participants) were designed to test for the plasticity of cognitive factors and causal links with symptom change in the short-term (within-session).

### Interventions promoting helpful attention patterns

#### Attention Bias modification interventions

**Fig. 3:**
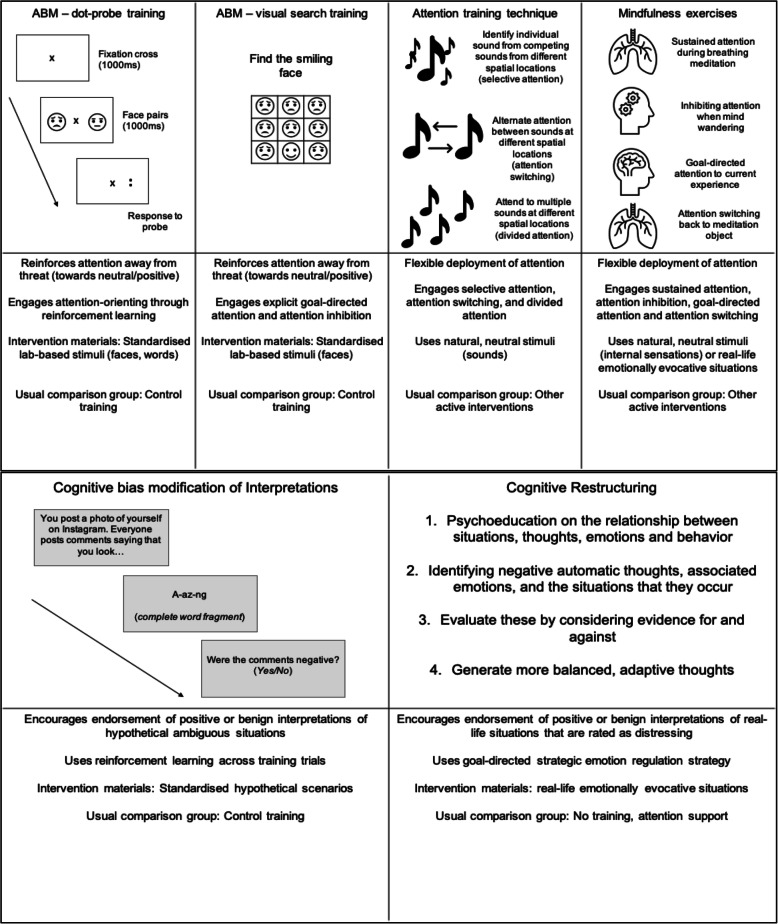
Summary of interventions promoting helpful attention (top panel) and interpretation patterns (bottom panel)

Of 22 attention studies [8–28], nineteen (86%) used Attention Bias Modification (ABM) training to shift (and reinforce) maladaptive attention patterns away from threatening/negative to neutral or positive stimuli across multiple training trials. Common training tasks are the *visual dot-probe* and *visual search training tasks* [29] (Fig. 3). The visual dot-probe (and its’ variants) train the orienting of attention away from threatening/negative cues towards neutral/positive cues, by presenting response-probes more frequently behind neutral/positive stimuli. The visual search task promotes goal-directed attention, by instructing participants to identify positive stimuli from an array of competing threatening/negative stimuli. Across studies, ABM training was delivered between 1 and 13 sessions. Most studies employed a similar computerised task not designed to modify attention as an active comparison condition. For the visual dot-probe, this control condition involved responding to a probe that appeared with equal frequency behind a threatening/negative versus neutral/positive stimuli. In the visual search control task, participants searched for a 5-petalled flower from 7-petalled flowers. Most studies delivered ABM via computers, but smartphone methods were also trialled [30].

Ten ABM studies reported significantly greater reduction of attention biases for threatening/negative information in the intervention than the comparison condition [8, 12, 17, 18, 20–24, 26]. Three other studies reported expected changes on attention bias but only when particular variants/versions of ABM were used (visual search [25], spatial cueing [23]); or under specific combinations of stimuli by exposure conditions [14].

#### Attention flexibility interventions

Two studies (27, 28, 9%) aimed to improve general Attention Flexibility (AF, Fig. 3) using the Attention Training Technique (ATT) [31] and/or a Mindfulness-based intervention. ATT is designed to strengthen the ability to flexibly use and control attention through explicit instruction [31]. Across training phases, participants engage in selective attention, attention switching and dividing their attention between neutral (e.g. sounds) stimuli in the environment. Mindfulness-based interventions can also target general attention regulation [32]. Exercises may benefit concentration (the sustained aspect of attention), effortful attention-inhibition of distracting information, goal-directed attention control, and flexible switching of attention. While the focus is usually on neutral stimuli or internal sensations, these exercises also involve ‘sitting with’ more unpleasant sensations (e.g. pain) in non-judgmental way.

Only one of the two studies targeting AF collected measures to assess changes in attention patterns [28]. Participants receiving ATT or mindfulness showed similarly large (significant) pre-to-post increases in questionnaire measures of attention flexibility.

### Symptom reduction effects of promoting helpful attention patterns

#### ABM interventions

Three multisession visual dot-probe ABM studies were conducted in clinical samples (Table 1). One involving young people with Social Anxiety Disorder [10] found small within-(intervention)-group reductions in symptoms from pre-to-post-intervention, but only ABM participants showed continued decreases to a 3-month follow-up. The absence of significant reductions in attention biases suggests that symptom changes were not driven by measurable changes in attentional patterns. Interestingly, this study noted that within adolescents allocated to receive ABM, those with higher *trait attention control* (reported by parents) showed significantly lower social anxiety symptoms at post-intervention. ABM was also assessed as a way of augmenting the effects of Cognitive Behavioural Therapy (CBT) in young people with complex forms of anxiety [9]. Those who received ABM showed large within-group symptom reduction with large differences to the control-training-plus-CBT group at post-intervention. The authors did not report scores on attention bias measures making it difficult to attribute large symptom reduction effects to changes in attention patterns. Working with young people with depression [8], one study reported large within-group symptom reduction in the intervention group and a large group difference with control participants post-intervention (effects that persisted to a 12-month follow-up). Importantly, there were greater reductions in attention bias scores among those receiving ABM than the control condition, suggesting that symptom change could be due to attention change.

**Table 1:**
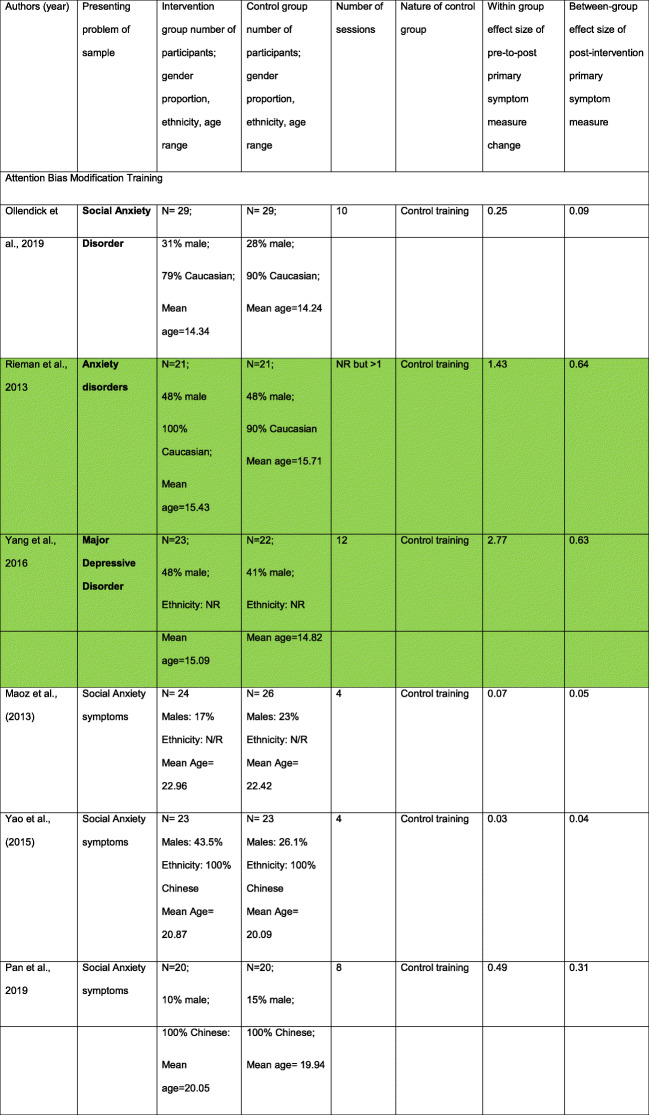
Promoting helpful attention patterns in participants with clinical symptoms (in bold) and high symptom scores (not bold). Where studies do not give separate demographic information for each group, these are combined in a single cell. Where effect sizes were not reported or could not be calculated, these are labelled as Not Reported (NR). Green highlighted rows reflect studies showing large within group symptom reduction and at least medium sized between group effects. Orange highlighted rows reflect studies showing near large within group symptom reduction and small sized or non-reported between group effects

Eleven ABM studies were conducted in high-symptom participants (Table 1). Seven involved young people with social or general anxiety symptoms [11–14, 16–18]. With one exception [14], symptom reduction effects from pre-to-post intervention in the ABM training condition were small across studies (Cohen’s d = 0.03–0.49). Two [20, 21] (of the 4) studies targeting depression reported moderate-to-large size reduction in symptoms in the ABM condition (at post-intervention and follow-up time-points) that were linked with significant changes in attention bias. However only one of these reported a significant difference to their comparison condition at post-training and follow-up assessments [20]. One study reported no differential reduction of depression symptoms in the ABM compared to control group [15], and unexpectedly, one study [19] found greater symptom decreases in control participants.

#### AF interventions

In a comparison of ATT with mindfulness in young people with high anxiety/depression symptoms [28], both groups showed medium-sized improvements, which were maintained at 6 months. Improvement in questionnaire reports of attention flexibility, significantly predicted treatment response. The second study [27] compared a mindfulness-based intervention with CBT in young people with depressive symptoms at-risk for Type 2 diabetes. Greater symptom reduction occurred in the mindfulness group than the CBT group at post-treatment (Cohen’s d = 0.56) and at 6 months follow-up (Cohen’s d = 0.69). No attention process measures were collected.

### Interventions promoting helpful interpretation patterns

#### Cognitive Bias modification of interpretations interventions

Fifty-two studies [17, 18, 27, 30, 33–81] modified interpretations. Twenty-three (44%) included Cognitive Bias Modification of Interpretations (CBM-I) training. Similar to ABM, this uses repeated reinforcement learning to encourage the endorsement of positive (or benign) interpretations of ambiguous information, over-riding the tendency to infer threatening/negative explanations. A common training task presents individuals with incomplete written ambiguous situations; completion of a word fragment resolves ambiguity in a positive/benign direction (Fig. 2). Most studies present incomplete emotionally-neutral situations as an active comparison condition. The number of training sessions across clinical, high-symptom and unselected studies ranged from single-sessions to 15.

Of 23 studies, 18 (78%) reported clear training effects in the CBM-I intervention condition, that is, either decreased negative interpretations or increased benign/positive interpretations from pre-to-post intervention or relative to the comparison condition at post-intervention. However, most studies used a measure of interpretational style that was structurally similar to the training task and could reflect demand effects. Where studies assessed transfer effects using a different measure of interpretation style, training effects in the CBM-I condition were mixed [33, 37, 54].

#### Cognitive restructuring interventions

Thirty-one studies (59%) used Cognitive Restructuring (CR). This uses explicit instruction (Fig. 2) to encourage individuals to generate alternative explanations for situations and to consider evidence for and against each explanation. CR is a routine component in many CBT protocols for youth anxiety/depression, but can be used as a standalone intervention. Varying between 8 and 16 sessions, CR can be administered in individual or group sessions, face-to-face or remotely. One study [74] used Cognitive Reappraisal training, a version of CR focused on teaching participants to re-interpret distressing situations, sometimes through a third-party perspective (“psychological distancing”).

Seven of the 31 studies collected measures to inform changes in cognitive patterns, but none directly measured interpretation style. Yet, all 7 showed expected changes in the intervention compared to the comparison condition, which could reflect the products of increased positive/benign interpretation of daily situations. One reported changes in adolescents’ estimation of certain anxiety-provoking events [40]. Another reported reductions in irrational beliefs [61]. Decreases in automatic negative thoughts and increases in automatic positive thoughts [52] as well as decreases in self-negative statements [46, 62], and increases in positive cognitions around hypothetical stressful situations [79] were reported.

### Symptom reduction effects of promoting helpful interpretation patterns

#### CBM-I interventions

Two studies (Table 2) delivered multisession CBM-I training to young people with clinical depression [33, 34], reporting small-to-moderate symptom reduction changes in the intervention group (Cohen’s d = 0.02/0.51). There were also small differences post-intervention with the control condition (Cohen’s d = 0.10/0.32). No studies delivered multi-session CBM-I training to young people meeting diagnostic criteria for an anxiety disorder.

**Table 2:**
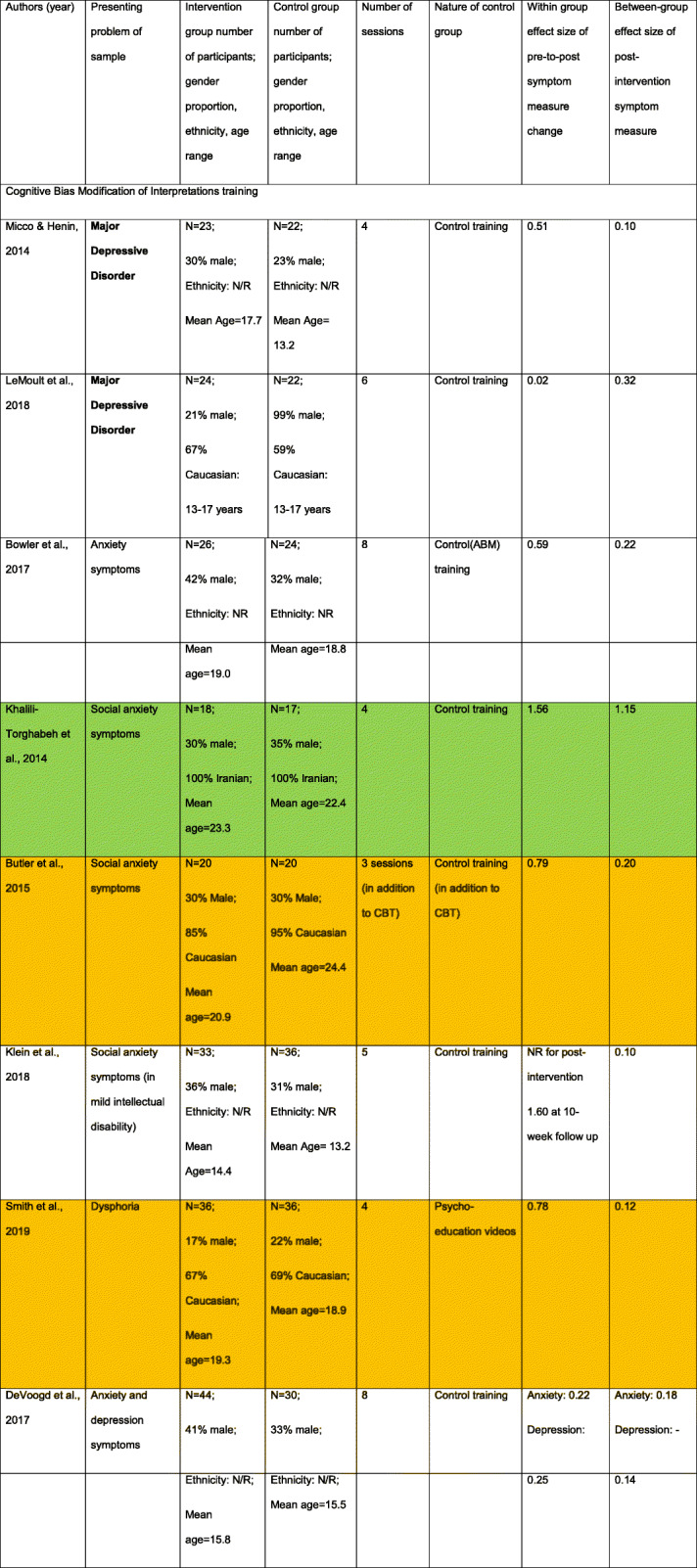
Promoting helpful interpretation patterns in participants with clinical symptoms (in bold) and high symptom scores (not bold). Where studies do not give separate demographic information for each group, these are combined in a single cell. Where effect sizes were not reported or could not be calculated, these are labelled as Not Reported (NR). Green highlighted rows reflect studies showing large within group symptom reduction and at least medium sized between group effects. Orange highlighted rows reflect studies showing near large within group symptom reduction and small sized or non-reported between group effects

Six studies applied CBM-I to young people with high symptom scores (Table 2). Only one reported large symptom improvement in the CBM-I group, and equally large differences compared to a control condition, with expected changes in interpretational style [35]. Three studies of general anxiety [18], social anxiety [36] and dysphoria [38] showed medium-sized symptom reduction in the CBM-I group (Cohen’s d = 0.59–0.79) and small between-group differences with various comparison conditions post-intervention (Cohen’s d = 0.12–0.22). Finally, two studies targeting social anxiety [37] or anxiety/depression [17] reported small within-group symptom reduction and small post-intervention differences with the comparison condition. However, for one, CBM-I training effects on symptoms were more apparent at 10-weeks (Cohen’s d = 1.60) [37], with expected changes in positive interpretation.

#### CR interventions

Seven studies assessed CR techniques within CBT in young people with clinical anxiety and/or depression, as the primary condition or co-morbid with PTSD (Table 2). Three were case series [39, 43, 81], where no data on symptom measures was reported, or were based on fewer than 5 participants. Two studies targeting anxiety disorders (social anxiety [41], panic [40]) showed large reductions in symptoms from pre-to-post intervention in the CR condition. Micco and colleagues [40] recorded session-to-session change on anxiety, and noted a therapeutic gain following the first session of CR. However, in both studies, there was either no data reported from the (wait-list) control group, or the control group was another active intervention (behavioural activation [41]), in which case the between-group difference at post-intervention on anxiety symptoms was small. Using CR to target depression, two studies revealed large within-group symptom reduction effects [42, 44], but only one employed a comparison condition (comprising monitoring and non-specific counselling), and reported a moderate sized post-intervention difference in depressive symptoms [42].

Nine studies employed CR (within CBT) in young people with high symptom levels. Two assessed CR effects on anxiety symptoms with one finding weak [45], and the other strong [46], within-group reduction effects. The study reporting the stronger within-group changes noted a reduction in negative self-statements [46]. A case series aiming to reduce anxiety symptoms in young people with a chronic health condition [47] did not report whole-sample mean changes but all 6 participants improved across treatment. For the 6 studies assessing depression symptoms (with one targeting depression in a sample at-risk for a chronic health condition [27]), 4 reported large within-intervention-group pre-to-post symptom reduction effects and medium-to-large between-group differences with a comparison condition (treatment as usual or attention support) at post-intervention [42, 48–50]. The two other studies reported weak or medium-sized symptom reduction effects [27, 51]; the one reporting smaller-sized changes assessed this at 4 months [51] so improvements may have become weak with time.

Nine studies examined standalone CR interventions in young people with high general anxiety/depression symptoms or with specific test, speech or performance anxiety (Table 2). Three noted significant reductions in anxiety measures from pre-to-post-intervention in the CR condition but did not report enough data to calculate effect sizes [58, 60, 61]. Where effect sizes were reported, within-group symptom reduction were medium to large (Cohen’s d = 0.72–2.43). Two studies that reported large effect sizes also found expected changes in automatic negative thoughts and negative/positive self-statements in the CR group [52, 62]. Comparisons with waitlist/no-intervention conditions across studies showed weak to large between-group differences at post-intervention. One study noted that individuals with *lower purposeful engagement* (the reduced tendency to attend/engage with unpleasant thoughts) benefited more from CR techniques than those receiving the comparison condition [44].

### Amplifying the effects of attention and interpretation interventions

#### Combined interventions

Six studies jointly targeted attention and interpretation patterns in reducing anxiety/depression. Four involved clinical participants. The first [82] delivered a web-based intervention combining CR and ATT in reducing social anxiety. Effect sizes (Cohen’s d) for the intervention group were 0.72–0.82 on symptoms from baseline to a 4-month follow-up, and were significantly greater than changes reported in the wait-list control group. Piet and colleagues [83] combined mindfulness-based cognitive therapy with CBT in socially-anxious young adults, thus targeting AF and CR. Combining interventions yielded greater within-group symptom reduction but this increase was marginal compared to receiving one treatment (Cohen’s d = 0.20–0.33). O’Toole and colleagues [84] applied Emotion Regulation Therapy to young people with generalised anxiety disorder, cultivating AF (shifting and sustaining attention on a difficult experience) and CR abilities. Within-intervention-group reductions emerged on anxiety symptoms (Cohen’s d = 1.2–1.4), preceded and mediated by changes in cognitive reappraisal and decentring, a cognitive skill, inversely correlated with negative self-referential processing. Finally, a one-day group-based CBT package was delivered to 24 young people with clinical anxiety/depression [85]. Of 8 workshop topics, one corresponded to CR and one to AF (within mindfulness). The study aimed to gather qualitative feedback from young people. One theme that emerged was that the wide selection of techniques enabled young people to learn a suitable technique.

Two case series [86, 87] piloted a combined ABM and CBM-I intervention to reduce high anxiety/depression symptoms. Neither included a comparison condition and neither was powered to detect significant within-group changes. The first showed reduction of social anxiety symptoms in around 80% of participants. Using a similar intervention but adapted/translated for young people with a history of victimisation in the UK and Nepal, social anxiety scores reduced only in UK participants (Cohen’s d = 0.81). Qualitative feedback across both studies suggested poor engagement with ABM than CBM-I training.

### Consultations with lived experience representatives

Young peoples’ perspectives on how thinking patterns could amplify negative emotions were used to support our research focus (Fig. 1). However, several more messages emerged from discussing the use of helpful cognitive patterns in daily life, barriers and how these could be enhanced.

First, participants spoke about adopting alternative perspectives, such as those used in CR or CBM-I, as being useful in daily life. One young person also noted that this was especially using a third-person perspective.

“*Yeah so you don’t think all the focus is on you … you could say something from a positive angle like they could be talking about somebody else not talking about you.*” 21-year old female, Asian British.

“*The therapy helped me by not having the negative thoughts.. making me aware of it … talking about this, is it really the person or is it me, or getting another perspective … rather than cutting the relationship has helped*” 24-year old female, White European.

“*Challenging those thoughts do [es] help, especially trying to find pieces of evidence to go against what I think … It is also helpful to get someone else to suggest alternative perspectives*” 21-year old female, Chinese.

However, some noted obstacles in current interventions, underscoring a need to help young people discover methods for learning and implementing helpful cognitive patterns:

“*In counselling, they try to think about what is and isn’t irrational. And try to think about all the positive explanations before you jump to negatives … if I can recognise it is happening this can be easier, but very often I don’t so it doesn’t help massively*”. 17-year old female, White British.

To improve their effectiveness, one young person suggested using *both* attention and interpretation patterns to manage negative emotions:

“*I think someone once told me that a thought only lasts for 8 seconds unless you chose to prolong it yourself. For me, I find it easier to engage it a little more and find out where it has come from, why do I think this, where has it come from, and then challenge the idea in my head*” 19-year old male, White British.

Young people also described that the deliberate recall of positive past experiences or positive aspects of oneself could help to counteract negative thinking:

“*May be if I try to point out the things that are good and maybe compare it to another piece of artwork I’ve done before. Or maybe think “well you’ve struggled on this but look now, you’ve managed to do this better than you’ve done before*”” 15-year old female, White British.

“*I write down things I appreciate about myself … Say if I cooked dinner for my friends, they didn’t give that much positive feedback, I might automatically assume my cooking wasn’t that good or I had not cooked enough food. But then if I wrote down that I actually cooked for them, I might feel better.*” 21-year old female, Chinese.

## Discussion

Helpful attention and interpretation patterns can potentially help young people better navigate daily-life emotional situations. As youth is a time in which maladaptive cognitive patterns and their links with anxiety/depression consolidate [88, 89], implementing more resilient cognitive responses can divert away from negative trajectories. Our search of the scientific literature revealed a myriad of techniques: these could be differentiated by whether they target attention or interpretation (or both) but they could also be divided into bias modification training techniques (ABM, CBM-I) versus instructed, strategic, goal-directed techniques. Our findings show interventive potential for each category whether targeting attention or interpretation or whether directed at clinical or sub-clinical symptoms. Here, we summarise these findings along with messages from consultations with young people and suggest necessary research to realise these areas of intervention potential, within this quickly expanding field.

### ABM and CBM-I techniques

Emerging from cognitive science research, bias modification training studies were designed to target a specific mechanism and its causal link with symptoms. Thus, studies usually include a well-matched comparison condition and pre to post-measures of the intended mechanism, enabling one to draw inferences over the cognitive origins of symptom reduction. Given these dedicated efforts to target the mechanism, ABM and CBM-I studies broadly showed changes in bias (although questions remain over the poor reliability of reaction time based measures of attention bias and transfer effects of training to other interpretation bias measures). Less observed were consistently medium or large reductions in symptoms either in clinical or high-symptom groups following ABM/CBM-I. However, these training paradigms were not designed to engage clients in multi-session interventions. Training is presented on a computer using lab-developed stimuli (faces, words) with no psychoeducation rationale for the intervention. Training dosage is somewhat arbitrarily determined and the infrequent inclusion of follow-up assessments makes it difficult to assess whether symptom changes occur only after consolidation of training.

To address these issues, adult and pre-adolescent ABM studies have begun to base new training paradigms on visual search protocols more, as these may more efficiently change maladaptive attention patterns by enhancing the voluntary capacity to select positive/benign stimuli [29]. Implementations have shown consistent symptom reduction [90, 91]. In adults, a version of the visual search training paired with reinforcement incentives (e.g. music that plays when looking at smiling faces over negative faces), has yielded strong training and symptom reduction effects in adults with social anxiety [92] and major depression [93]. A further advantage is the use of eye-tracking to monitor and measure gaze patterns, yielding more reliable and valid assessments of attention patterns across time. While this promising “gaze-contingent music reward therapy” has been trialled in pre-adolescents [94], it has not been assessed in young adults. For CBM-I, symptom reduction effects could be augmented by tailoring materials to the day-to-day lives of the targeted population to increase engagement and generalisation to real life. Adult research also suggests the use of prolonged imagery to self-generate outcomes during training to scaffold training effects [95]. Harnessing virtual reality within training could also be fruitful in engaging young people [96]. If we are to discover the clinical potential of these mechanism-based training interventions, it will be crucial to address issues around user-engagement, co-designing these with young people.

### Attention flexibility and cognitive restructuring interventions

Unlike bias modification training, AF and CR techniques have developed over decades, as part of complex cognitive-behavioural interventions. These use naturalistic stimuli or real-life scenarios to embellish learning, are guided by clinical insights over dosage, and evaluated as part of larger trials with lfollow-up assessments, increasing the opportunity to demonstrate clinical effects. Here, both AF and CR interventions were associated with consistent medium/large symptom reduction effects in across clinical and high-symptom participants. However, as these broader complex intervention programmes target many other treatment components, use either no or poorly matched comparison groups and often do not include direct measures of attention and interpretation patterns, it is difficult to attribute symptom reduction to changes in cognitive patterns. Isolating “active ingredients” of symptom reduction is important for developing brief, accessible, scalable and transportable interventions. Thus, the next wave of research should focus on delineating the mechanisms underlying these interventions. Grounding these within rigorous experimental studies that use active comparison conditions to control for other non-specific variables and routine inclusion of pre and post-intervention measures of cognitive variables is important.

## Conclusions and next steps

Once developed, an urgent question is: how best to deliver these techniques (dosage, mode) and to whom? Few studies have assessed individual differences in intervention responsiveness. Some assessed whether baseline symptoms moderated improvements but findings across studies were inconsistent [10, 19]. A handful of studies investigated individual differences in attention control [10] or purposeful engagement [64] but these findings require replication. Rather than personally tailoring interventions, an alternative approach is to develop an universal “toolkit” for young people; this could open up the possibility of having multiple techniques to choose from, which young people find appealing [85]. Consistent with this, and the combined cognitive bias hypothesis [5, 6], targeting several biases in interventions could yield benefits “greater than the sum of their parts”. Combined approaches could be promising. Young people we consulted suggested that combining techniques to target attention and interpretation patterns could be helpful. Other strategies (e.g. recalling positive experiences) to amplify the use of helpful attention or interpretation patterns could also be beneficial. Delivering explicit instructions that encourage changes in strategic cognitive processes with reinforcement training to target more habitual ways of responding, could leverage interactive benefits across different levels of responding. For such multi-target, multi-level interventions to be sustainable, these will need co-design and co-evaluation with young people themselves. In addition, one lived experience representative suggested that a lack of recognition or understanding could affect usage of implementing these strategies. Therefore, it may also be useful to include psychoeducation or mental health literacy principles within these interventions, in order to help young people recognise the role of unhelpful thinking styles in everyday life and why there is a need to target these through helpful strategies.

## Data Availability

The datasets used and/or analysed during the current study are available from the corresponding author on reasonable request.
